# Food sources for the Ediacara biota communities

**DOI:** 10.1038/s41467-020-15063-9

**Published:** 2020-03-09

**Authors:** Ilya Bobrovskiy, Janet M. Hope, Elena Golubkova, Jochen J. Brocks

**Affiliations:** 1 Research School of Earth Sciences, Australian National University, Canberra, ACT Australia; 2 Institute of Precambrian Geology and Geochronology, Russian Academy of Sciences, St. Petersburg, Russia; 3 Present Address: Division of Geological and Planetary Sciences, California Institute of Technology, Pasadena, CA USA

**Keywords:** Coevolution, Palaeontology, Carbon cycle, Geochemistry

## Abstract

The Ediacara biota represents the first complex macroscopic organisms in the geological record, foreshadowing the radiation of eumetazoan animals in the Cambrian explosion. However, little is known about the contingencies that lead to their emergence, including the possible roles of nutrient availability and the quality of food sources. Here we present information on primary producers in the Ediacaran based on biomarker molecules that were extracted from sediments hosting Ediacaran macrofossils. High relative abundances of algal steranes over bacterial hopanes suggest that the Ediacara biota inhabited nutrient replete environments with an abundance of algal food sources comparable to Phanerozoic ecosystems. Thus, organisms of the Ediacara biota inhabited nutrient-rich environments akin to those that later fuelled the Cambrian explosion.

## Abstract (web summary)

Complex macroscopic organisms are first found in the Ediacaran period, but their ecology during this time is not well understood. Here, Bobrovskiy et al. analyse biomarkers from Ediacaran sediments hosting macrofossils and find evidence for abundant algal food sources available for these organisms.

## Introduction

The appearance of the first macroscopic, morphologically complex organisms in the late Ediacaran (~571–541 million years [Ma] ago) was the onset of one of the most important biological transitions in Earth’s history. However, it remains unclear which environmental, ecological and evolutionary factors accompanied the emergence of large animal-like organisms, and how these various factors interacted. The most popular models connect the appearance and ecological success of animals with rising atmospheric oxygen concentrations in the Neoproterozoic^1–3^. Yet, molecular oxygen as a limiting resource faces several challenges. While increasing oxygenation of deep marine waters in the Neoproterozoic broadly coincides with the appearance of macroorganisms in such environments^4^, suitably oxic conditions were probably established in well aerated surface environments hundreds of millions of years earlier^5–9^. Moreover, cyanobacterial mats are ubiquitous in shallow-marine environments in the Proterozoic and would have provided stable ecospace with elevated oxygen concentrations for organisms with mat-related lifestyles far back in Earth history^10,11^.

An alternative environmental factor that may have constrained the timing of eumetazoan emergence and radiation is nutrient availability, including bio-limiting elements and efficient carbon sources supplied by primary producers. The abundance of different classes of primary producers in Precambrian oceans can be tracked using molecular fossils, or biomarkers^12–14^. The most common biomarkers are alteration products of membrane lipids such as hopanols found in numerous aerobic bacteria, including cyanobacteria, and sterols produced by algae and other eukaryotes. During sedimentary diagenesis, hopanols are transformed into hydrocarbon hopanes, while sterols yield fossil steranes. Hopanes and steranes are extracted from ancient sedimentary rocks using organic solvents and quantified using gas chromatograph-mass spectroscopy. The proportion of hopanes (H) over steranes (S), the H/S ratio, can be used as an estimate for the relative flux of bacterial versus eukaryotic organic matter to bottom sediments, and thus as a first-order approximation for the relative importance of algal versus cyanobacterial biomass, among both benthos and plankton^12–14^. The transition from bacteria- to eukaryote-dominated primary production in the oceans, recorded by biomarkers, occurred ~650-635 Ma^15^ ago and may have been crucial for the success of most macroscopic heterotrophs^15,16^.

**Fig. 1:**
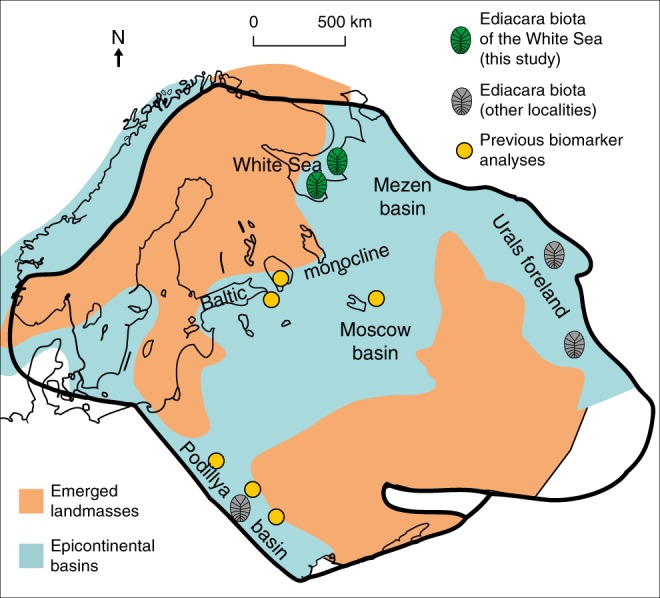
Map contrasting the fossil and biomarker localities of this (green) and previous (orange) biomarker studies at the East European Platform. The grey symbols indicate known localities of the Ediacara biota where sediments are too thermally mature to preserve biomarkers. Modified after Pehr et al.^17^ and Sliaupa et al.^50^.

The Ediacara biota represents the first global appearance of large heterotrophic organisms, including animals, in the fossil record, and thus reflects aspects of the ecology and evolution of early animals. Recent studies have reported biomarkers from Ediacaran sediments of the East European Platform (EEP) with H/S ratios that are high relative to typical Phanerozoic marine conditions^17,18^. Consequently, it has been suggested that Ediacaran macroorganisms preferentially inhabited oligotrophic environments and used bacteria as their main energy source, as either food or symbionts^17,19–21^. It is notable, however, that none of the biomarker data came from localities that preserve Ediacaran macrofossils (Fig. 1), and it remains to be seen whether they usefully reflect ecological circumstances across the whole of the EEP. In order to resolve the particular circumstances under which Ediacaran macroorganisms lived, we analyse biomarkers from sediments with Ediacaran macrofossils from the White Sea area of the EEP, and show that eukaryotic algae were abundant among the food sources available for the Ediacara biota.

## Results and Discussion

### Geological context of Ediacaran deposits of the White Sea area

Due to the mild thermal history of Ediacaran deposits in the White Sea area, Ediacaran macrofossils co-occur there with extremely well preserved biomarkers^22,23^, offering a unique opportunity to assess the habitat and ecological preferences of these problematic organisms. The White Sea area offers localities with the most diverse and abundant Ediacara biota in the world, including various members of the White Sea, Avalon and Nama assemblages^24–27^. All sediments from the Lyamtsa and Zimnie Gory localities (Fig. 1) were originally deposited in shallow-marine environments and preserve evidence for persistent and pervasive microbial mats^28^.

The distribution of Ediacaran macrofossils within the White Sea area sections is largely controlled by taphonomy—the fossils are best preserved at the soles of sandstone layers, but less abundant fossils of poorer quality are scattered throughout the entire sedimentary succession^25,27^. To get a full picture about the distribution of primary producers in depositional environments associated with the Ediacara biota, samples collected from the entire section exposed in the studied localities were analysed for biomarkers (including 52 samples from fossiliferous intervals and six samples from adjacent stratigraphic levels devoid of fossils). Most organic geochemical studies analyse centimetre-scale sedimentary rock samples, thus averaging ecological signals across substantial periods of time, and potentially missing ecologically distinct endmembers. To ensure that we indeed capture the exact ecological environment of organisms of the Ediacara biota, we analysed sediments immediately beneath and above surfaces with Ediacara biota fossils at millimetre resolution. As organisms of the Ediacara biota are preserved in situ, biomarkers extracted from clay underneath the fossils represent the substrate they were living on. Sandstones above fossils normally represent storm deposited material^28^; biomarkers extracted from these sandstones presumably average the biomass of the local environment, but may also contain material transported from adjacent settings. Specifically, we analysed two surfaces in the Lyamtsa locality that contain abundant *Dickinsonia*, *Parvancorina* and *Palaeopascichnus* fossils, and well-studied surfaces in the Zimnie Gory, known as Z1(I) and Z11(XXII), which contain *Andiva*, *Archaeaspinus, Armilifera*, *Aspidella*, *Brachina, Charniodiscus*, *Cyanorus*, *Cyclomedusa, Dickinsonia*, *Inaria*, *Ivovicia*, *Kimberella*, *Onega*, *Ovatoscutum*, *Paleophragmodictya*, *Paravendia*, *Parvancorina*, *Tamga*, *Temnoxa*, *Tribrachidium* and *Yorgia*^27^.

Thus, this study looks at biomarkers representative of environments for a broad range of species of the Ediacara biota with variable feeding strategies, including burrowers (e.g. *Sabellidita*, *Calyptrina*)^29,30^, mat-scrapers (e.g. *Andiva*, *Dickinsonia, Kimberella*) and potentially filter- or osmotroph-feeding mat stickers (Arborea, e.g. *Charniodiscus*). It was impossible to obtain biomarker data from particular sections and time intervals that only contain a single assemblage of the Ediacara biota^24^ due to their high thermal maturity (e.g. Newfoundland, United Kingdom, or Namibian sections). However, allowing for some extrapolation, the data collected in the current study provide information about the local ecological environment of representative organisms of all three existing Ediacara biota assemblages, which otherwise occur in a wider temporal and palaeogeographic context.

### Hopane/Sterane ratios and primary producers from the Tonian to Phanerozoic

**Fig. 2:**
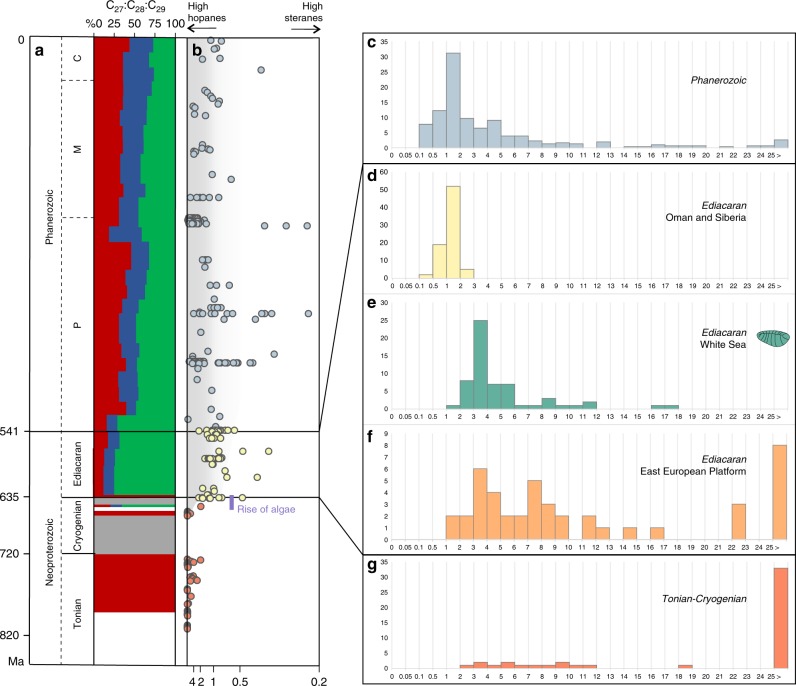
Timeline through the Neoproterozoic and Phanerozoic and abundance information for hopanes and steranes. **a** The relative abundance of the sterane homologues cholestane (C_27_, green), ergostane (C_28_, blue) and stigmastane (C_29_, green). Size of the coloured areas reflects relative sterane abundances. **b** Evolution of the relative abundance of bacterial hopanes over eukaryotic steranes (H/S) through time (orange = Tonian and Cryogenian; yellow = Ediacaran (data from Oman and Siberia); blue = Phanerozoic); the scale goes from H/S ~ ∞ (with no steranes detected) to H/S = 0.25, converted from the S/H ratio values from 0 to 4 in Brocks et al.^15^. Histograms showing the abundance distribution of H/S values for different periods and locations: **c** Phanerozoic biomarker data reported in the literature; **d** Ediacaran of the South Oman Salt Basin and the Siberian platform; **e** Ediacaran of the White Sea area; **f** previous biomarker analyses on the Ediacaran of the EEP (localities are marked as orange circles on Fig. 1); **g** Tonian and Cryogenian biomarker data reported in the literature. Data from the EEP from Pehr et al.^17^, data for the White Sea this study, all other data recalculated from Brocks et al.^15^.

To place the Ediacaran biomarkers from the White Sea area into a broad temporal context, Fig. 2 summarizes data for bacterial hopanes and algal steranes from the Tonian to the present. Based on biomarkers, bacteria were the only notable primary producers in Paleo- and Mesoproterozoic oceans^31^. Primitive eukaryotic sterane signatures emerged ~900–800 Ma ago^15,32^, although the overall H/S ratio remained high (70% of values are H/S > 29.5, and steranes are below detection limits in 43% of samples that contain hopanes). Moreover, it is unclear whether steranes in Tonian sediments are derived from algae or other organisms^15^. The first signs of unambiguous algal productivity occurred in a single sample assigned to the Cryogenian, around 650 Ma, and started to be a dominating signal from the very beginning of the Ediacaran, close to 635 Ma^15,33^. This ‘rise of algae’ was marked by an order-of-magnitude drop in H/S ratios and the emergence of a near-modern sterane diversity (Fig. 2a, b). Phanerozoic sediments, in contrast to the Tonian, mainly demonstrate continuously low H/S values, (H/S = 0.5–5 range accounts for 70% percentile with the mode at 1.3), indicating that algae have been key primary producers for the past 541 million years.

The Ediacaran, however, yields bitumens with H/S signatures resembling both the Tonian and Phanerozoic. Sediments from the Oman Salt Basin and the Siberian platform demonstrate H/S values similar to the Phanerozoic (Fig. 2c, d) (H/S = 0.75–1.4 range accounts for 70% percentile with the mode value 1.3). By contrast, bitumens from the Ediacaran interior seaway of the EEP^17^ include numerous elevated H/S values up to 119 (H/S = 1.5–12.5 range accounts for 70% percentile with several modes, and median value 7.7), values otherwise typical for the Tonian (Fig. 2f). It has been suggested^17^ that the elevated H/S values in the interior seaway of the EEP, just as those from the Tonian^15,16^, might reflect a strong predominance of bacteria among primary producers, potentially due to regionally oligotrophic conditions, thus highlighting a high spatial heterogeneity in primary producer communities in Ediacaran marine environments (though see Supplementary Note 2)^17^.

Based on the H/S data from global Ediacaran sediments, the Ediacara biota might have either inhabited newly established algae-rich environments^15,16^ or, conversely, thrived in nutrient-depleted ecosystems dominated by bacterial primary productivity akin to the Tonian or Mesoproterozoic^17,20^. If latter hypothesis is correct, then the diet and ecology of organisms of the Ediacara biota must have been very distinct from most Phanerozoic animals^17,19,20^, and it would invalidate the premise that the emergence of abundant algal food sources was crucial for the ecological success of eumetazoan animals. Biomarker data from sediments directly associated with Ediacara biota fossils can shed light on this dispute.

### Primary producers at the Ediacara biota localities

**Fig. 3:**
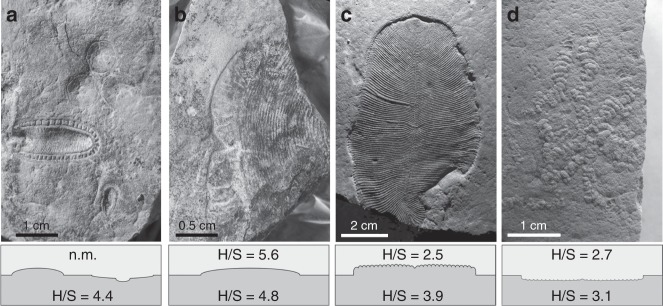
Examples of Ediacaran macrofossils from the White Sea Area and associated H/S ratios from sediments immediately over- and underlying the fossils. **a** *Aspidella* and *Kimberella* from the Z11(XXII) surface (Zimnie Gory, photo by E. Uryvaeva); **b** *Andiva* from the Z1(1) surface (Zimnie Gory), the surface also contains *Archaeaspinus*, *Armilifera*, *Brachina*, *Charniodiscus*, *Cyanorus*, *Cyclomedusa*, *Dickinsonia*, *Inaria*, *Ivovicia*, *Kimberella*, *Onega*, *Paravendia*, *Parvancorina*, *Temnoxa*, *Tribrachidium*, *Yorgia*^27^; **c** *Dickinsonia* (Lyamtsa); **d** *Palaeopascichnus* (Lyamtsa). n.m., not measurable.

The clay and sandstone material directly surrounding Ediacara biota fossils in the White Sea sections demonstrate low H/S ratios (H/S = 2.5–5) (Fig. 3). The overall H/S values from the whole set of White Sea area sediments vary slightly more broadly (H/S = 1–7 range accounts for 70% percentile with the mode value 3.3, *n* = 59; Fig. 2e). The H/S value for the only sample that has been reported from the Ediacara biota localities in Arctic Siberia (H/S = 2.7)^34^ falls near the mode value for the White Sea area, although it is impossible to judge whether this sample is representative of the whole section. These H/S values suggest that the Ediacara biota inhabited environments with near-modern fluxes of bacterial versus eukaryotic biomass (Fig. 3). Moreover, steranes of the White Sea area are strongly dominated by stigmastanes (C_29_ (%) = 75 ± 9% of C_27_–C_29_ sterane homologues; *n* = 59). The C_29_ predominance among steranes in the White Sea area is characteristic of Ediacaran biomarker signatures and likely reflects predominance of green algae among eukaryotes^35^. The mode of H/S value in the White Sea area is somewhat elevated in comparison to the Phanerozoic (Fig. 2c, e). This elevation may reflect a stronger relative contribution of planktonic cyanobacterial biomass than the average Phanerozoic, but is more likely associated with benthic cyanobacterial mats that are widespread in White Sea sediments and generally abundant in Ediacaran shallow-water environments^36,37^. Regardless, Ediacaran macroorganisms of the White Sea area inhabited environments that were orders of magnitude enriched in algal relative to bacterial food sources when compared with the Tonian, and in this respect similar to Phanerozoic marine habitats.

### Primary producers and early animal evolution

The rise of algae 650-635 Ma has changed the ecosystems on our planet forever, although the trigger for this phenomenon is unclear. It has been proposed that a potential increase in nutrient levels in the oceans at that time could have made algae more competitive relative to photosynthetic bacteria^15,16^, or alternatively that protistan eukaryotes or bacteriovorous sponge-grade animals effectively reduced bacterial biomass, thus providing ecospace for algae^6,7,33,38–40^. Whatever the causes for the proliferation of planktonic algae, their biomass may have fuelled the radiation of eumetazoan animals by increasing the efficiency of nutrient and energy transfer to higher trophic levels based on larger cells sizes compared with bacterial phytoplankton, and by supplying fast sinking food particles to benthic animal communities at the sea floor^15,16^. However, this is to ignore that microbial mats were present in the oceans long before algal food became available. With the discovery of a motile lifestyle among the earliest branching Eumetazoa (Placozoa and Ctenophora^41^), pervasive cyanobacterial mat coverage in well-oxygenated shallow-water environments would have provided ample resources for metazoan proliferation. Yet, the vast majority of extant eumetazoan animals prefers a eukaryote-dominated diet; with rare exceptions, eumetazoans are not sustained by purely bacterial food sources^42,43^. A preference for a eukaryotic diet may thus be the ancestral state of Eumetazoa, possibly due to the high nutritional quality of algal biomass.

Based on sedimentological evidence^28^ and biomarker data presented here, the Ediacara biota of the EEP inhabited well-mixed oxygenated shallow-water environments with levels of potential algal food supply comparable to the Phanerozoic. This observation in the White Sea area is consistent with a recent spatial analyses of ~570 Ma old Ediacaran ecosystems on the Avalon Peninsula, overlapping with the White Sea area taxonomically, that showed that competition for resources was not the driving factor for local Ediacaran communities^44^. Unlike previously predicted^17,19,20^, bacteria-dominated ecosystems in Ediacaran marine basins were generally not a cause of the unusual appearance and ecology of the Ediacara biota. Rather, the ecological and evolutionary bridge leading from a mid-Proterozoic bacteria-dominated world to the appearance of Phanerozoic animal-dominated ecosystems was paved with algae-rich environments akin to those preferred by modern eumetazoan animals. Although morphologically and possibly phylogenetically distinct from Phanerozoic animals, the large organisms of the Ediacara biota were a part of newly established nutrient and energy-rich environments that later hosted the Cambrian diversification of animal life.

## Methods

### Sample collection

Samples were collected during fieldworks in the White Sea region (Russia) in 2015–2017 specifically for biomarker analysis. Samples are coming from around 40 m thick stratigraphic interval of Lyamtsa and Arkhangelsk Beds (Ust-Pinega Formation, Redkino Regional Stage) in the Lyamtsa Ediacara biota locality, and from around 120 m thick interval of Vaysitsa, Zimnie Gory (Ust-Pinega Formation, Redkino Regional Stage) and Erga beds (Mezen Formation, Kotlin Regional Stage) in the Zimnie Gory locality. Samples were collected avoiding weathered zones and cracks and were immediately wrapped in pre-baked aluminium foil (300 °C, 9 h) and packed in calico bags under strict avoidance of contamination.

Deposits in the Lyamtsa and Zimnie Gory localities of the Ediacara biota are mainly represented by clay and mm-scale heterolithic interlamination of clay, siltstone and sandstone, with occasional 0.5–10 cm, sometimes up to 60 cm thick sandstone lenses and layers. In the Zimnie Gory locality, nearly every siltstone and sandstone, including mm-thick lenses in the interlamination, has a microbial mat impression at the base; in Lyamtsa, these impressions are relatively rarer, and some surfaces are erosional. Fossils are typically preserved at the base of sandstone layers with microbial mat impressions, however some species are also sometimes found within clay^23,45^. Samples include sediments immediately (within the first millimetres) underlying (clay) and overlying (typically sandstone) surfaces with Ediacara biota fossils, as well as 59 samples from sedimentary layers throughout the exposed sections that are not known to contain fossils. Ediacaran macroorganisms are found in situ, therefore biomarker composition of clays they were living on was taken as the closest approximation of environments the Ediacara biota inhabited. Sandstones overlying the fossils are mostly of storm origin^28^ and would contain reworked organic matter deposited in the environments with the Ediacara biota, but might also contain material transported from other environments.

### Sample preparation and extraction

Samples were prepared in an ultra-clean facility dedicated to trace biomarker analysis at the ANU. To identify and eliminate any surficial trace contaminants, the clay was analysed using the so-called exterior–interior protocol^46–48^. The outer 3–4 mm of the sample were removed using a micro drill (Dremel® 400 Series Digital, Mexico) with a combusted (290 °C, 9 h) and solvent cleaned saw nozzle and represented the exterior (‘E’) portion. Analysis of the last saw-blade solvent rinse confirmed that it was free of detectable contaminants. The exterior and the interior portions of the clay sample were ground to powder (>240 mesh) using a steel puck mill (Rocklabs Ltd, Onehunga, New Zealand). The mill was cleaned using dichloromethane and methanol, and by grinding combusted (600 °C, 9 h) quartz sand. As the samples were collected from outcrops into aluminium foil and cotton bags, which has been proven to prevent contamination^23^, for ten samples both interior and exterior portions were analysed as control; for the rest, we analysed only interior portions, but exterior portions were kept in case there is any contamination in the analysed exterior portions or increased maturity parameters in these samples.

Bitumen was extracted from rock powder using an accelerated solvent extractor (ASE 200, Dionex, USA) with DCM: methanol (9:1), reduced to 100 µl under a stream of nitrogen gas. All solvents used in the study were 99.9% grade (UltimAR^®^; Mallinckrodt Chemicals, St. Louis, MO, USA); all glassware was cleaned by combustion at 300 °C for 9 h. An extract was then fractionated into saturated, aromatic and polar fractions using micro-column chromatography over annealed (300 °C; 12 h) and dry packed silica gel (Silica Gel 60; 230– 600 mesh; EM Science). Saturated hydrocarbons were eluted with 1 dead volume (DV) of *n*-hexane, aromatic hydrocarbons with 4.5 DV of *n*-hexane: DCM (1:1) and the polar fraction with 3 ml DCM: methanol (1:1). An internal standard, 18-MEAME (18-methyleicosanoic acid methylester; Chiron Laboratories AS), was added to the saturated and aromatic fractions for quantification. The samples were analysed and quantified by gas chromatography–mass spectrometry (GC–MS).

### Gas chromatography–mass spectrometry (GC–MS)

GC–MS analyses were carried out on an Agilent 6890 gas chromatograph coupled to a Micromass Autospec Premier double sector mass spectrometer (Waters Corporation, Milford, MA, USA). The GC was equipped with a 60 m DB-5 MS capillary column (0.25 mm i.d., 0.25 μm film thickness; Agilent JW Scientific, Agilent Technologies, Santa Clara, CA, USA), and helium was used as the carrier gas at a constant flow of 1 ml min^−1^. Samples were injected in splitless mode into a Gerstel PTV injector at 60 °C (held for 0.1 min) and heated at 260 °C min^−1^ to 300 °C. For full scan, selected ion recording and metastable reaction monitoring (MRM) analyses, the GC oven was programmed from 60 °C (held for 4 min) to 315 °C at 4 °C min^−1^, with total run time of 100 min. All samples were injected in *n*-hexane to avoid deterioration of chromatographic signals by FeCl_2_ build-up in the MS ion source through use of halogenated solvents^49^. Steranes were quantified in MRM mode in M^+^ → 217 and hopanes in M^+^ → 191 transitions relative to the C_27_ Tm hopane isomer content. The C_27_ Tm hopane isomer content was quantified in a full-scan mode (*m*/*z* 191) relative to the 18-MEAME internal standard (*m*/*z* 340). Peak areas are uncorrected for differences in GC–MS response. Quantification errors were calculated as a function of a peak area established for the GC–MS used in this study^22^.

### Laboratory system blanks

Comprehensive, accumulatory system blanks were performed covering all analytical steps including extraction, fractionation and instrumental analysis. For this purpose, a combusted (600 °C, 9 h) sand was ground to powder and extracted using the methodologies and identical tools described above.

### Reporting summary

Further information on research design is available in the Nature Research Reporting Summary linked to this article.

## Supplementary information

Supplementary Information

Reporting Summary

## Data Availability

All data required to understand and assess the conclusions of this research are available in the main text and supplementary materials. Rock samples, extracts and digital raw GC–MS data are stored at the Australian National University.
