# Supplementary material for: Food sources for the Ediacara biota communities
Source: Nat Commun. 2020 Mar 9;11:1261. doi: 10.1038/s41467-020-15063-9 (PMC7062841; doi:10.1038/s41467-020-15063-9)
Supplement: Supplementary file 1 — Supplementary Information [file 41467_2020_15063_MOESM1_ESM.pdf]

Supplementary Information

Food sources for the Ediacara biota communities

Bobrovskiy et al.

Supplementary Note 1: Syngeneity of biomarkers

GC-MS MRM, SIR and full-scan analyses of the comprehensive accumulatory laboratory system blanks confirmed that the detected hydrocarbons were not introduced by laboratory processes. Monitoring of the blanks yielded no *n*-alkanes, hopanes or steranes, even when measured using the most sensitive GC-MS MRM methods. The only contaminants detected in the blanks are trace amounts of phthalates—plasticizers present in small amounts in solvents used for extraction of biomarkers.

The high βα/(βα + αβ) and low Ts/(Ts +Tm) hopane isomer ratios, virtual absence of diasteranes and αββ sterane isomers[1,2], which are only found in the most immature sediments, indicate that the hydrocarbons in the Ediacaran deposits in the White Sea area are significantly below the oil-generative window, which means that the kerogen never thermally generated and expelled liquid hydrocarbons[3]. Such a low thermal maturity is never observed in contaminant petroleum products and migrated oils, which by their very nature must have a maturity within the oil generative window. This makes it very easy to track contamination by simply assessing the maturity parameters[2,4].

The results of Exterior/Interior (E/I) experiment for all analysed samples show nearly identical concentrations of all biomarkers in exterior and interior rock portions, with E/I values ranging from 0.75 to 2.35 for different compounds (Supplementary Table 2), which is within the range on natural variability within samples and indicates that the precombusted aluminium foil and clean calico bags prevented contamination of samples during transportation and storage, and confirm that all detected compounds are indigenous[2].

Supplementary Note 2: High hopane/sterane ratio values in the Ediacaran of the EEP

The unusually high H/S ratios reported from the interior of EEP have been interpreted as reflecting a strong predominance of bacteria among primary producers in the basin[5], thus providing evidence for high heterogeneity in the distribution of major groups of photosynthetic organisms in the Ediacaran. However, while steroids and hopanoids have similar preservation potential in the sedimentary record due to the similarity of their structure[6], it cannot be completely excluded that these high H/S values may have been caused by severe bacterial degradation of eukaryotic biomass, causing replacement of algal sterols with hopanols from heterotrophic bacteria. An extreme case of such severe degradation in modern environments is, for instance, observed in soils, where primary plant debris may become so severely recycled by the soil microbiome that H/S rises to >> $10^{3,7,8}$. If this is the case, the local differences in H/S in the EEP are caused by different taphonomic conditions; if true, this would indicate that algae were already prevailing primary producers in the Late Ediacaran across the globe.

Supplementary Table 1. Relative distribution of hopanes and steranes in the White Sea area Ediacaran deposits in stratigraphic order, *EB – intervals highlighted in green indicate intervals with fossils of the Ediacara biota; †H/S = $\Sigma(C_{27\text{-}35}$ hopanes) / $\Sigma(C_{27\text{-}29}$ steranes), hopanes: $C_{27} = \Sigma$(Ts, Tm, β), $C_{29} = \Sigma$(αβ, Ts, βα), $C_{30} = \Sigma$(αβ, βα), $C_{31\text{-}35} = \Sigma$(αβ-22(S+R), βα), αβ = 17α(H)21β(H), βα = 17β(H)21α(H); steranes: $C_{27} = \Sigma$(βα-20(S+R)-diacholestane, ααα- and βαα-20(S+R)-cholestane), $C_{28} = \Sigma$(βα-20(S+R)-diaergostane, ααα- and βαα-20(S+R)-ergostane), C_{29} = Σ(βα-20(S+R)-diastigmastane, ααα- and βαα-20(S+R)-stigmastane), ααα = 5α(H),14α(H),17α(H), βαα = 5β(H),14α(H),17α(H); ‡C_{27} (%) = 100 * C_{27} steranes / $\Sigma(C_{27\text{-}29}$ steranes); §C_{28} (%) = 100 * C_{28} steranes / $\Sigma(C_{27\text{-}29}$ steranes); ||C_{29} (%) = 100 * C_{29} steranes / $\Sigma(C_{27\text{-}29}$ steranes); ¶thin interlamination of clay, siltstone and sandstone; #numbers in parentheses are standard deviation values calculated for the instrumental error based on repeat injections of a standard (see Methods), value 0.0 indicates that the standard deviation is less than 0.05; the green horizontal lines highlight sediment surfaces with abundant fossils of the Ediacara biota where the sediments immediately above and below where investigated for biomarkers (Fig. 3).

Locality	Regional stage	Beds	EB*	Sample	Lithology	H/S†	C_{27} (%)‡	C_{28} (%)§	C_{29} (%)‖
Zimnie Gory	Kotlin	Erga		**z2-16-5**	thin interlamination¶	16.9 *(0.0)*#	35.1 *(0.4)*	10.5 *(0.2)*	54.4 *(0.7)*
				**2-14-15**	clay	2.6 *(0.0)*	28.5 *(1.0)*	13.8 *(0.8)*	57.8 *(3.2)*
				**z2-14-4**	thin interlamination	5.4 *(0.8)*	15.4 *(3.0)*	11.7 *(1.2)*	72.9 *(4.4)*
				**z2-13-6**	thin interlamination	3.5 *(0.2)*	20.4 *(0.9)*	13.1 *(0.7)*	66.5 *(3.2)*
				**z2-13-4**	thin interlamination	4.9 *(0.0)*	23.6 *(0.3)*	11.1 *(0.2)*	65.3 *(0.8)*
				**z2-13-1**	sandstone	11.2 *(0.0)*	23.7 *(0.2)*	14.5 *(0.1)*	61.7 *(0.5)*
				**z2-12-2**	thin interlamination	5.0 *(0.3)*	21.5 *(1.1)*	12.5 *(0.8)*	65.9 *(2.4)*
				**z4-2-3**	sandstone	5.6 *(0.0)*	38.0 *(0.3)*	9.7 *(0.2)*	52.3 *(0.6)*
				**z4-1-2**	clay	4.8 *(0.0)*	23.4 *(0.4)*	9.6 *(0.2)*	67.0 *(1.1)*
				**z4-1-4**	clay	5.6 *(0.0)*	27.7 *(0.4)*	8.7 *(0.2)*	63.5 *(0.7)*
				**z4-1-1**	clay	4.8 *(0.1)*	23.4 *(0.7)*	9.6 *(0.3)*	67.0 *(1.3)*
				**z2-9-4**	thin interlamination	4.4 *(0.1)*	19.2 *(0.7)*	8.9 *(0.2)*	72.0 *(1.2)*
				**z2-9-2**	thin interlamination	5.5 *(0.0)*	29.8 *(0.3)*	10.8 *(0.2)*	59.4 *(0.9)*
	Redkino	Zimnie Gory		**z2-7-4B**	thin interlamination	8.3 *(0.0)*	27.6 *(0.1)*	9 *(0.0)*	63.3 *(0.6)*
				**z2-7-4A**	thin interlamination	8.8 *(0.0)*	27.3 *(0.2)*	8.2 *(0.1)*	64.5 *(0.6)*
				**z2-6-10**	thin interlamination	4.3 *(0.1)*	21.0 *(0.6)*	8.6 *(0.4)*	70.4 *(1.3)*
				**z2-6-7**	clay	3.5 *(0.5)*	11.8 *(1.4)*	9.4 *(0.6)*	78.8 *(2.1)*
				**z2-6-6**	thin interlamination	4.5 *(0.2)*	22.6 *(1.0)*	10.2 *(0.4)*	67.2 *(1.8)*
				**z2-6-5**	sandstone	8.6 *(0.1)*	22.0 *(0.4)*	12.7 *(0.2)*	65.3 *(1.0)*
				**z2-5-4**	clay	17.5 *(0.1)*	32.6 *(0.5)*	13.1 *(0.3)*	54.3 *(1.2)*
				**z2-5-1**	clay	8.0 *(0.0)*	28.2 *(0.2)*	9.0 *(0.1)*	62.8 *(0.6)*
				**z2-3-6**	thin interlamination	10.1 *(0.1)*	22.2 *(0.5)*	10 *(0.3)*	67.8 *(1.2)*
				**z2-3-3**	sandstone	11.6 *(0.1)*	21.3 *(0.2)*	11.2 *(0.1)*	67.5 *(1.1)*
				**z2-3-2**	thin interlamination	3.9 *(0.1)*	19.5 *(0.4)*	10.8 *(0.3)*	69.7 *(1.6)*
				**z2-3-1**	thin interlamination	5.8 *(0.0)*	21.7 *(0.3)*	11.1 *(0.2)*	67.1 *(1.2)*
				**z2-2-1**	clay	5.9 *(0.1)*	9.9 *(1.0)*	5.7 *(0.3)*	84.4 *(1.3)*
				**z2-1-8**	sandstone	3.3 *(0.0)*	12.7 *(0.3)*	10.3 *(0.1)*	76.9 *(0.6)*
				**z2-1-5**	clay	3 *(0.1)*	11.1 *(0.5)*	7.7 *(0.3)*	81.2 *(1.3)*
				**z6-8-1**	thin interlamination	6.8 *(0.0)*	36.2 *(0.3)*	10.5 *(0.1)*	53.3 *(0.8)*
				**z6-6-2**	clay	1.7 *(0.2)*	18.5 *(0.9)*	9.7 *(0.5)*	71.9 *(1.8)*
				**z6-4-3**	thin interlamination	3.3 *(0.0)*	13.3 *(0.2)*	10.3 *(0.1)*	76.4 *(0.6)*
				**z6-4-2**	clay	3.6 *(0.0)*	11.9 *(0.5)*	9.4 *(0.3)*	78.7 *(1.3)*
				**z6-1-1**	clay	3.2 *(0.0)*	21.5 *(0.3)*	17.7 *(0.2)*	60.8 *(1.2)*
		Vaysitsa		**z5-k1**	sandstone	9.9 *(0.1)*	27.2 *(0.8)*	11.7 *(0.2)*	61.1 *(2.5)*

Lyamtsa	Redkino	Arkhangelsk		**31-3-3**	clay	4.1 *(0.0)*	9.8 *(0.0)*	8.0 *(0.0)*	82.3 *(0.0)*
		Lyamtsa		**31-1-6m**	thin interlamination	3.7 *(0.1)*	11.2 *(0.4)*	7.7 *(0.3)*	81.1 *(2.0)*
				**31-1-5m**	thin interlamination	3.0 *(0.0)*	13.1 *(0.2)*	9.1 *(0.2)*	77.9 *(1.0)*
				**35-4-3**	clay	3.3 *(0.0)*	8.4 *(0.2)*	7.3 *(0.2)*	84.3 *(0.9)*
				**35-2-9m**	sandstone	2.7 *(0.0)*	10.2 *(0.2)*	8.3 *(0.2)*	81.5 *(1.4)*
				**35-2-8m**	thin interlamination	3.1 *(0.0)*	10.0 *(0.2)*	8.0 *(0.2)*	82.0 *(1.1)*
				**35-1-5m**	thin interlamination	3.1 *(0.0)*	10.4 *(0.2)*	7.9 *(0.1)*	81.7 *(0.9)*
				**35-1-3m**	thin interlamination	3.1 *(0.0)*	10.9 *(0.2)*	7.1 *(0.2)*	82.0 *(1.1)*
				**35-02-1m**	thin interlamination	3.3 *(0.0)*	11 *(0.2)*	8.1 *(0.1)*	81.0 *(1.0)*
				**35-01-2m**	sandstone	2.3 *(0.0)*	11.3 *(0.3)*	8.6 *(0.2)*	80.1 *(1.2)*
				**35-01-1m**	clay	3.3 *(0.0)*	10.8 *(0.2)*	8.1 *(0.2)*	81.2 *(1.0)*
				**D-Ly S**	sandstone	2.5 *(0.0)*	11.7 *(0.2)*	8.6 *(0.1)*	79.7 *(0.9)*
				**D-Ly C**	clay	3.9 *(0.0)*	9.7 *(0.2)*	7.9 *(0.2)*	82.4 *(1.1)*
				**34-5-4m**	thin interlamination	3.1 *(0.0)*	10.3 *(0.2)*	8.0 *(0.1)*	81.7 *(0.9)*
				**34-4-4m**	thin interlamination	3.1 *(0.0)*	10.6 *(0.3)*	8.1 *(0.2)*	81.3 *(1.3)*
				**34-4-2m**	thin interlamination	3.4 *(0.1)*	12.2 *(0.4)*	8.2 *(0.3)*	79.6 *(2.2)*
				**34-5-2 in**	clay	3.3 *(0.0)*	10.6 *(0.3)*	7.7 *(0.2)*	81.7 *(1.2)*
				**34-3-4m**	thin interlamination	3.2 *(0.0)*	11.6 *(0.3)*	8.4 *(0.2)*	80.0 *(1.4)*
				**34-2-3m**	thin interlamination	3.2 *(0.0)*	10.1 *(0.3)*	8.1 *(0.2)*	81.8 *(1.2)*
				**34-2-2m**	sandstone	2.9 *(0.0)*	11.3 *(0.2)*	8.6 *(0.2)*	80.1 *(1.1)*
				**34-01-MN**	thin interlamination	2.9 *(0.0)*	11.1 *(0.3)*	7.5 *(0.2)*	81.3 *(1.5)*
				**34-01-2**	clay	3.3 *(0.0)*	9.1 *(0.2)*	7.1 *(0.1)*	83.8 *(0.8)*
				**34-01-7m**	thin interlamination	2.5 *(0.0)*	11.2 *(0.2)*	8.3 *(0.1)*	80.5 *(0.9)*
				**34-01-6m**	thin interlamination	3.0 *(0.0)*	9.9 *(0.0)*	7.7 *(0.0)*	82.4 *(0.0)*

Supplementary Table 2. Results of the 'Exterior/Interior' experiments for hopanes and steranes in selected samples, *relative proportion of a compound concentrations in the exterior and the interior portions of a sample; †Dia/Reg I – proportion of diastigmastanes of the total stigmastanes in the interior portions; ‡Dia/Reg E – proportion of diastigmastanes of the total stigmastanes in the exterior portions; §Mor/Hop I - proportion of βα (moretanes) of the total C_{27} hopanes in the interior portions; ||Mor/Hop E - proportion of βα (moretanes) of the total C_{27} hopanes in the exterior portions.

Sample	E/I*				Maturity parameters			
	Hopanes	C_{27} steranes	C_{28} steranes	C_{29} steranes	Dia/Reg I†	Dia/Reg E‡	Mor/Hop I§	Mor/Hop E||
**z4-1-2**	1.04 *(0.01)*	0.97 *(0.03)*	1.13 *(0.04)*	0.92 *(0.02)*	0.04 *(0.00)*	0.05 *(0.00)*	0.33 *(0.01)*	0.33 *(0.01)*
**z6-4-2**	0.75 *(0.01)*	0.76 *(0.03)*	0.79 *(0.04)*	0.78 *(0.03)*	0.04 *(0.00)*	0.05 *(0.01)*	0.33 *(0.01)*	0.32 *(0.02)*
**z6-8-1**	1.54 *(0.02)*	2.35 *(0.07)*	1.56 *(0.05)*	1.6 *(0.03)*	0.21 *(0.00)*	0.14 *(0.00)*	0.23 *(0.01)*	0.31 *(0.01)*
**z6-4-3**	1.07 *(0.03)*	1.35 *(0.12)*	1.33 *(0.12)*	1.14 *(0.06)*	0.20 *(0.00)*	0.18 *(0.00)*	0.23 *(0.01)*	0.22 *(0.01)*
**31-3-3**	0.90 *(0.02)*	1.37 *(0.09)*	1.19 *(0.08)*	1.05 *(0.04)*	0.05 *(0.02)*	0.05 *(0.01)*	0.27 *(0.02)*	0.26 *(0.02)*
**35-01-2m**	0.83 *(0.01)*	0.82 *(0.02)*	0.79 *(0.02)*	0.71 *(0.01)*	0.05 *(0.00)*	0.05 *(0.00)*	0.26 *(0.01)*	0.25 *(0.01)*
**34-5-2**	1.12 *(0.02)*	1.90 *(0.12)*	2.04 *(0.14)*	1.28 *(0.05)*	0.04 *(0.01)*	0.06 *(0.00)*	0.30 *(0.01)*	0.26 *(0.01)*
**34-2-3m**	0.67 *(0.01)*	0.80 *(0.02)*	0.77 *(0.02)*	0.71 *(0.01)*	0.05 *(0.00)*	0.06 *(0.00)*	0.31 *(0.01)*	0.26 *(0.02)*
**34-01-MN**	0.94 *(0.01)*	1.05 *(0.05)*	1.04 *(0.04)*	1.05 *(0.02)*	0.05 *(0.00)*	0.05 *(0.00)*	0.25 *(0.01)*	0.25 *(0.01)*

Supplementary References

1 Bobrovskiy, I. *et al.* Ancient steroids establish the Ediacaran fossil *Dickinsonia* as one of the earliest animals. *Science* **361**, 1246-1249, doi:10.1126/science.aat7228 (2018).

2 Bobrovskiy, I., Hope, J. M., Krasnova, A., Ivantsov, A. & Brocks, J. J. Molecular fossils from organically preserved Ediacara biota reveal cyanobacterial origin for *Beltanelliformis*. *Nature Ecology & Evolution* **2**, 437-440, doi:10.1038/s41559-017-0438-6 (2018).

3 Peters, K. E., Walters, C. C. & Moldowan, J. M. *The biomarker guide*. Vol. 2 (Cambridge University Press, 2005).

4 Goryl, M., Marynowski, L., Brocks, J. J., Bobrovskiy, I. & Derkowski, A. Exceptional preservation of hopanoid and steroid biomarkers in Ediacaran sedimentary rocks of the East European Craton. *Precambrian Research* **316**, 38-47, doi:https://doi.org/10.1016/j.precamres.2018.07.026 (2018).

5 Pehr, K. *et al.* Ediacara biota flourished in oligotrophic and bacterially dominated marine environments across Baltica. *Nature communications* **9**, 1807 (2018).

6 Peters, K., Walters, C. & Moldowan, J. *The Biomarker Guide. Volume 2. Biomarkers and Isotopes in Petroleum Systems and Earth History*. 2nd edn, (Cambridge University Press, 2005).

7 Ries-Kautt, M. & Albrecht, P. Hopane-derived triterpenoids in soils. *Chemical Geology* **76**, 143-151 (1989).

8 Moldowan, J. M., Seifert, W. K. & Gallegos, E. J. Relationship between petroleum composition and depositional environment of petroleum source rocks. *AAPG bulletin* **69**, 1255-1268 (1985).
