# Supplementary material for: Food sources for the Ediacara biota communities
Source: Nat Commun. 2020 Mar 9;11:1261. doi: 10.1038/s41467-020-15063-9 (PMC7062841; doi:10.1038/s41467-020-15063-9)
Supplement: Supplementary file 2 — Reporting Summary [file 41467_2020_15063_MOESM2_ESM.pdf]

nature research

Corresponding author(s): Ilya Bobrovskiy, Jochen Brocks

Last updated by author(s): Feb 10, 2020

Reporting Summary

Nature Research wishes to improve the reproducibility of the work that we publish. This form provides structure for consistency and transparency in reporting. For further information on Nature Research policies, see Authors & Referees and the Editorial Policy Checklist .

Statistics

For all statistical analyses, confirm that the following items are present in the figure legend, table legend, main text, or Methods section.

n/a	Confirmed	
☐	☒	The exact sample size (*n*) for each experimental group/condition, given as a discrete number and unit of measurement
☒	☐	A statement on whether measurements were taken from distinct samples or whether the same sample was measured repeatedly
☒	☐	The statistical test(s) used AND whether they are one- or two-sided<br>*Only common tests should be described solely by name; describe more complex techniques in the Methods section.*
☒	☐	A description of all covariates tested
☒	☐	A description of any assumptions or corrections, such as tests of normality and adjustment for multiple comparisons
☒	☐	A full description of the statistical parameters including central tendency (e.g. means) or other basic estimates (e.g. regression coefficient) AND variation (e.g. standard deviation) or associated estimates of uncertainty (e.g. confidence intervals)
☒	☐	For null hypothesis testing, the test statistic (e.g. *F*, *t*, *r*) with confidence intervals, effect sizes, degrees of freedom and *P* value noted<br>*Give P values as exact values whenever suitable.*
☒	☐	For Bayesian analysis, information on the choice of priors and Markov chain Monte Carlo settings
☒	☐	For hierarchical and complex designs, identification of the appropriate level for tests and full reporting of outcomes
☒	☐	Estimates of effect sizes (e.g. Cohen's *d*, Pearson's *r*), indicating how they were calculated

Our web collection on statistics for biologists contains articles on many of the points above.

Software and code

Policy information about availability of computer code

Data collection	n/a
Data analysis	n/a

For manuscripts utilizing custom algorithms or software that are central to the research but not yet described in published literature, software must be made available to editors/reviewers. We strongly encourage code deposition in a community repository (e.g. GitHub). See the Nature Research guidelines for submitting code & software for further information.

Data

Policy information about availability of data

All manuscripts must include a data availability statement. This statement should provide the following information, where applicable:

- Accession codes, unique identifiers, or web links for publicly available datasets
- A list of figures that have associated raw data
- A description of any restrictions on data availability

All data that supports the paper is presented in the main text of the manuscript and in the supplementary information. All raw biomarker data is deposited on servers of the Australian National University and accessible upon request. There are no formal restrictions on data availability.

Field-specific reporting

Please select the one below that is the best fit for your research. If you are not sure, read the appropriate sections before making your selection.

☐ Life sciences ☐ Behavioural & social sciences ☒ Ecological, evolutionary & environmental sciences

Ecological, evolutionary & environmental sciences study design

All studies must disclose on these points even when the disclosure is negative.

Study description	Biomarker analysis on Ediacaran deposits from the White Sea region (Russia)
Research sample	Ediacaran samples from the Lyamtsa and Zimnie Gory localities of the Ediacara biota in the White Sea region (Russia)
Sampling strategy	No sample-size calculation was performed
Data collection	Samples were collected by Ilya Bobrovskiy during field work 2015 to 2017.
Timing and spatial scale	No time-dependent measurements were made
Data exclusions	Weathered samples were not studied
Reproducibility	All attempts to repeat the experiment were successful.
Randomization	Samples were not allocated into groups.
Blinding	No statistical analyses were performed.

Did the study involve field work? [x] Yes [ ] No

Field work, collection and transport

Field conditions	Rock outcrops in the cliffs of the White Sea.
Location	Lyamtsa Village and Zimnie Gory in the Arkhangelsk Region (Russia)
Access and import/export	N/A
Disturbance	Only rock outcrops with no vegetation were explored.

Reporting for specific materials, systems and methods

We require information from authors about some types of materials, experimental systems and methods used in many studies. Here, indicate whether each material, system or method listed is relevant to your study. If you are not sure if a list item applies to your research, read the appropriate section before selecting a response.

Materials & experimental systems

n/a	Involved in the study	
[x]	[ ]	Antibodies
[x]	[ ]	Eukaryotic cell lines
[x]	[ ]	Palaeontology
[x]	[ ]	Animals and other organisms
[x]	[ ]	Human research participants
[x]	[ ]	Clinical data

Methods

n/a	Involved in the study	
[x]	[ ]	ChIP-seq
[x]	[ ]	Flow cytometry
[x]	[ ]	MRI-based neuroimaging
